# Microhabitat characteristics of the critically endangered big‐headed turtle *Platysternon megacephalum*

**DOI:** 10.1002/ece3.10633

**Published:** 2023-10-20

**Authors:** Fanrong Xiao, Rongping Bu, Zihao Ye, Jichao Wang, Hai‐Tao Shi

**Affiliations:** ^1^ Ministry of Education Key Laboratory for Ecology of Tropical Islands, Key Laboratory of Tropical Animal and Plant Ecology of Hainan Province, College of Life Sciences Hainan Normal University Haikou China

**Keywords:** biodiversity, food abundance, freshwater turtle, human disturbances, rocky substrate, stream habitat

## Abstract

Understanding the microhabitat requirements of an animal is vital for ensuring the success of targeted conservation and microhabitat restoration measures. The big‐headed turtle (*Platysternon megacephalum*) is a freshwater species that is distributed across Southeast Asia. Owing to the human threats posed by illegal pet trade and overharvesting for food and medicinal purposes, the species has undergone rapid decline. However, in Hainan, their microhabitat characteristics are still unknown, which is neither conducive to the conservation of the species nor to the establishment of the Hainan Tropical Rainforest National Park. This study examined the microhabitat characteristics of *P. megacephalum* using sample plot methods in the Diaoluo Mountain area of Hainan Tropical Rainforest National Park. Our results indicated that *P. megacephalum* prefers stream microhabitats with rocky substrates, several caves, and a high diversity of food sources. Microhabitat characteristics did not differ significantly between adults and juveniles. Our results suggest that protecting microhabitats and main food sources is important for the conservation of *P. megacephalum*. Our findings provide a reference for the protection of this species in Jianfeng Ridge, Yingge Ridge, and other areas in Hainan Tropical Rainforest National Park.

## Abstract 2

This study examined the microhabitat characteristics of *P. megacephalum* using cage and sample plot methods in the Diaoluo Mountain area of the Hainan Tropical Rainforest National Park. Our results indicated that big‐headed turtles prefer stream microhabitats at higher altitudes, in proximity to rocky substrates, several caves, and high diversity of food sources. Microhabitat characteristics did not differ significantly between adults and juveniles.

## 1 INTRODUCTION

Hainan Tropical Rainforest National Park has a diverse range of freshwater turtles, harboring 12 species or approximately one‐third of the total number in the country (Bu et al., 2023; Farkas et al., 2019; Shi et al., 2011). Among these, the big‐headed turtle (*Platysternon megacephalum*) is listed as one of the most endangered freshwater turtles in the Convention on International Trade in Endangered Species of Wild Fauna and Flora (CITES) appendix I (CITES, 2023) and as critically endangered by the International Union for Conservation of Nature (IUCN; Fong et al., 2021). In China, the species is listed as a national Class II protected species (National Forestry and Grassland Administration of China, 2021). *Platysternon megacephalum* is a monotypic species both at the genus and family level (Turtle Taxonomy Working Group et al., 2021).

Big‐headed turtles mostly inhabit the streams of the Diaoluo, Limu, and Wuzhi mountains, and the Jianfeng and Yingge ridge areas on Hainan Island (Shi et al., 2011); thus, this freshwater species is an important indicator species for assessing water system health in Hainan Tropical Rainforest National Park because it only occurs in unpolluted water bodies. However, there have only been studies on population density (Xiao et al., 2021a) and homing behavior (Xiao et al., 2021b) of the big‐headed turtle in the Diaoluo Mountain area. To date, there have been no studies on the characteristics of its microhabitat in Hainan.

A suitable microhabitat provides animals with the resources necessary to support their survival and reproduction (Nieuwolt, 1996). The study of the microhabitats of endangered species provides critical knowledge that can be used to develop protection measures and practical guidance for their ex‐situ conservation and resource management. However, studies on big‐headed turtle microhabitat characteristics in China have been conducted only in Henan (Shen et al., 2010), Guangdong (Wang, 2010), and Hong Kong (Sung et al., 2015), with the first two studies suffering from the complications of non‐native experimental individuals and small sample sizes, respectively. Thus, the present study investigates the microhabitat characteristics of the big‐headed turtle, aiming to provide ecological data that can be used for conservation and management planning in Hainan Tropical Rainforest National Park.

## 2 MATERIALS AND METHODS

Nine streams were selected in the Diaoluo Mountain area of Hainan Tropical Rainforest National Park (513.7–1050 m above sea level) as our study sites. A survey of freshwater turtles was carried out from 2015 to 2016 and from 2018 to 2019 at each site during summer and autumn, using the cage method. Random sampling was carried out by using isometric sampling methods. A total of 81 cages containing salted fish as bait were placed at 30‐m intervals along the streams (Xiao et al., 2021a). In areas with cliffs or other obstacles, cages were placed at 60‐m intervals. The cages were left for 3–5 days and checked every morning. The sex and age of each turtle were documented, with the differentiation between adults and juveniles being a carapace length of greater or less than 100 mm (Xiao et al., 2021a), respectively. Adults in which the cloaca extends beyond the posterior margin of the carapace are males. All cages were removed after the experiment, and the turtles were returned to their original capture locations.

A sample plot of 10 m in length and the width corresponding to the stream width (1.6–6 m) was established. A capture plot was defined as a sample plot with a capture cage containing a turtle at the center and the plot length extending 5 m up and down the stream. A no‐capture plot was defined as a sample plot centered around a cage with no turtles present. Ten ecological factors were recorded, namely vegetation coverage (%), water surface width (m), water depth (cm), water surface velocity (m/s), substrate type (stone/sand/mud, covering >60% of the plot), exposed stone rate (piece/m^2^), number of caves, plant root system (clump), food diversity (number of species in the turtle cage), and distance from anthropogenic disturbance (<50, 50–100, >100 m). Exposed stone rate refers to the number of stones protruding from the water surface per square meter of water. A cave was defined as a small cavity formed by two or more stones large enough to accommodate a big‐headed turtle nestled on the riverbed or the gap at the bottom of a large rock. Plant roots are potential hiding places for the big‐headed turtle and were recorded as the number of visible root clumps at the edge of the riverbank within the sample plot. Species such as invertebrates and fish caught in each cage were assumed to be prey for the turtles, which could easily enter the cage but escape with difficulty. The distance from anthropogenic disturbance was recorded as the distance from the cage to the nearest road in multiples of 50 m.

All data were analyzed using SPSS v.16 software (SPSS Inc). A Chi‐square test was used to analyze differences between (1) plots with adults or juveniles and (2) capture and no‐capture plots for two ecological factors: substrate type and distance from anthropogenic disturbance. A *t*‐test was used to test for statistical differences between capture and no‐capture plots and the two age classes in reference to the remaining eight ecological factors: vegetation coverage, water depth, water surface width, number of caves, food diversity, water velocity, exposed stone rate, and plant root system.

All fieldwork was conducted in strict accordance with the guidelines of the Animal Research Ethics Committee of Hainan Provincial Education Centre for Ecology and Environment, Hainan Normal University (HNECEE‐2014‐002), which conform to the law of the People's Republic of China.

## 3 RESULTS

**FIGURE 1:**
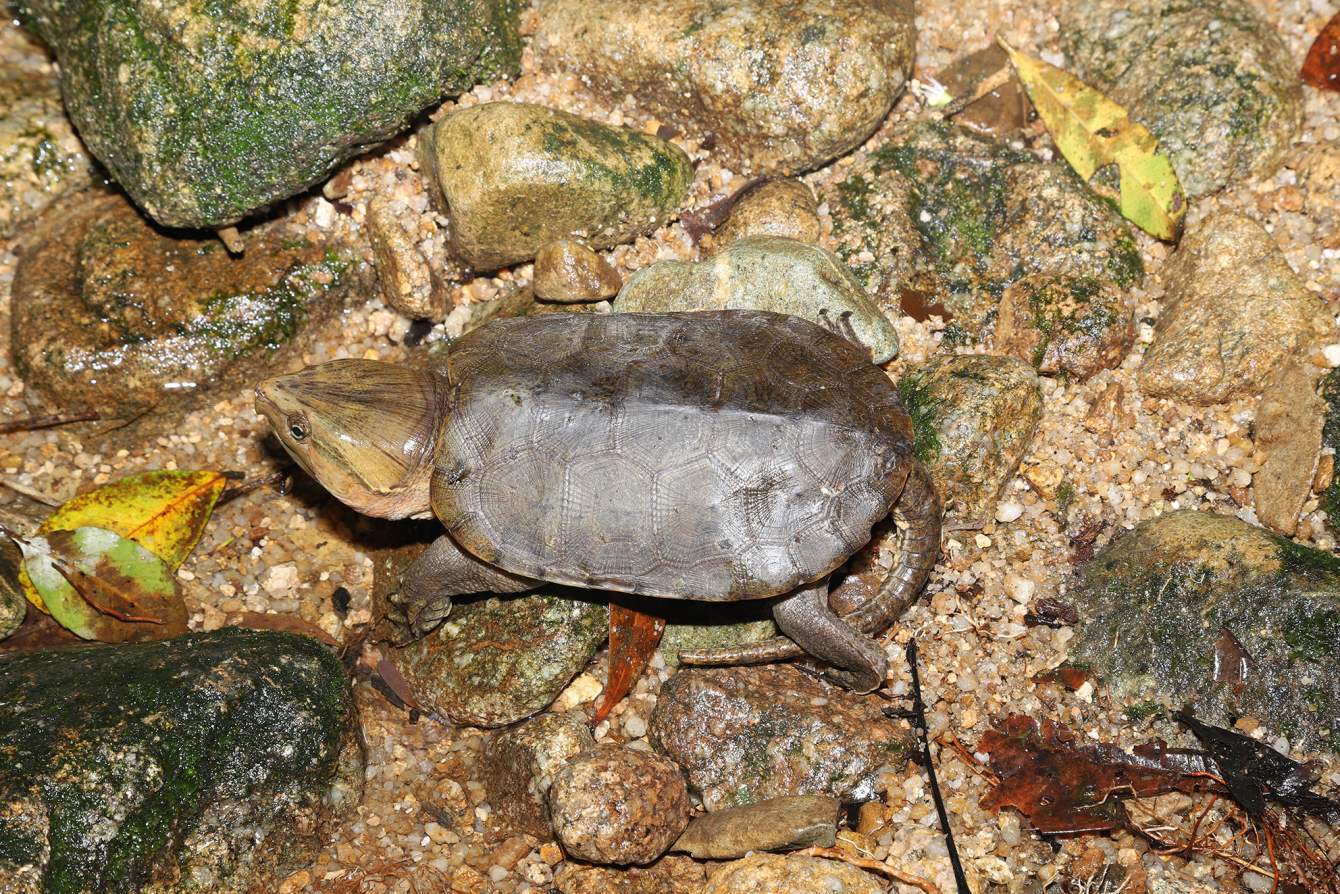
The big‐headed turtle *Platysternon megacephalum* and its stream microhabitat with rocky substrate. Photograph taken by Fanrong Xiao.

We observed 11 big‐headed turtles (Figure 1), comprising three adult females, three adult males, and five juveniles. All individuals were captured in streams at elevations ranging from 900 to 999 m. A total of 48 no‐capture plots and 13 capture plots were allocated from the 61 cages set within the elevation range of 900 to 999 m. The adult and juvenile turtles occupied 10 and 4 plots, respectively, of which one adult and one juvenile shared the same capture plot, and one juvenile was not recorded using the capture plot.

A comparison of the capture plots revealed no significant difference between adult and juvenile big‐headed turtles in the microhabitat characteristics (substrate type: *χ* ^2^ = 1.938, df = 1, *p* = .164; distance from anthropogenic disturbance: *χ* ^2^ = 2.869, df = 2, *p* = .238; Table 1).

**TABLE 1:** Microhabitat characteristics of adult and juvenile big‐headed turtles.

Ecological factor	Plot with adults (*n* = 10)	Plot with juveniles (*n* = 4)	*t‐*Test	*p*
Vegetation coverage (%)	0.61 ± 0.07	0.61 ± 0.12	−0.018	.986
Water depth (cm)	48.90 ± 4.62	41.75 ± 4.50	0.901	.385
Water surface width (m)	2.76 ± 0.27	2.42 ± 0.44	0.653	.524
Number of caves	14.20 ± 3.07	7.25 ± 3.01	1.316	.213
Food diversity (no. of species)	2.80 ± 0.13	3.00 ± 0.00	−0.926	.290
Water velocity (m/s)	0.08 ± 0.03	0.02 ± 0.02	1.116	.286
Exposed stone rate (piece/m^2^)	0.19 ± 0.07	0.04 ± 0.02	2.062	.064
Plant root system (clump)	2.4 ± 1.06	2.25 ± 1.31	0.8	.938

*Note*: Values indicate the means ± SE.

**FIGURE 2:**
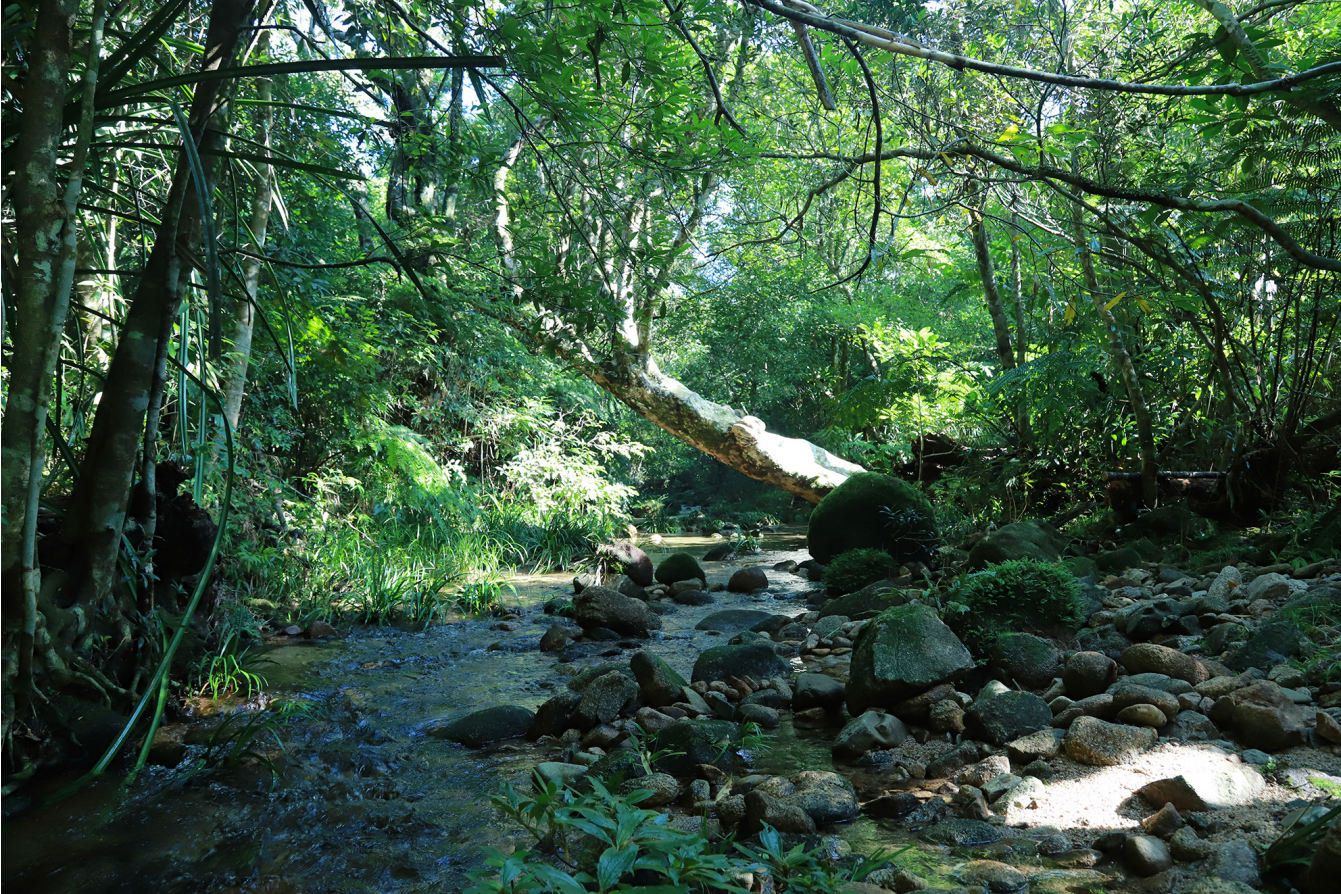
Vegetation along the stream microhabitat. Photograph taken by Fanrong Xiao.

Further comparison of all capture plots to the no‐capture plots showed that the big‐headed turtle preferred stream microhabitats with a rocky substrate (92.31%; *χ* ^2^ = 14.346, df = 2, *p* = .001), several caves, and diverse food (Table 2). Distance from anthropogenic disturbance (*χ* ^2^ = 3.41, df = 2, *p* = .182), vegetation coverage, water depth, water surface width, surface velocity, exposed stone rate, and plant root system showed no significant difference between the capture plots and no‐capture plots (Table 2; Figure 2).

**TABLE 2:** Big‐headed turtle microhabitat characteristics in no‐capture plots and capture plots.

Ecological factor	No‐capture plots (*n* = 48)	Capture plots (*n* = 13)	*t‐*Test	*p*
Vegetation coverage (%)	0.63 ± 0.03	0.62 ± 0.06	0.156	.876
Water depth (cm)	42.67 ± 2.91	47.84 ± 3.69	−0.874	.386
Water surface width (m)	3.13 ± 0.16	2.66 ± 0.87	1.434	.157
Number of caves	6.04 ± 0.78	12.46 ± 2.64	−2.336	.035
Food diversity (no. of species)	2.35 ± 0.12	2.85 ± 0.10	−3.138	.003
Water velocity (m/s)	0.06 ± 0.01	0.06 ± 0.03	−0.186	.853
Exposed stone rate (piece/m^2^)	0.08 ± 0.03	0.15 ± 0.06	−1.264	.214
Plant root system (clump)	2.19 ± 0.34	2.54 ± 0.86	−0.441	.661

*Note*: Values indicate the means ± SE.

## 4 DISCUSSION

Our results showed that big‐headed turtles are often found in stream microhabitats with rocky substrates, several caves, and a variety of food. Our finding is consistent with previous research findings which reported that big‐headed turtles primarily occupy streams and only visit terrestrial habitats during the female breeding season (Shen et al., 2010). Caves that have formed on the riverbed can provide important refuges from predators for big‐headed turtles. This phenomenon has been observed in a study by Wang (2010), who found that big‐headed turtles preferred microhabitats with rocky substrates and more than 18 caves. Other stream‐dwelling turtle species, such as the four‐eyed spotted turtle (*Sacalia quadriocellata*) and Beal's eyed turtle (*S. bealei*), have also been found to prefer rocky substrates (Gong et al., 2005; Hu et al., 2016). Furthermore, the shape and color of the big‐headed turtle's carapace resemble river stones; thus, rocky microhabitats may provide camouflage, as observed in *S. quadriocellata* (Xiao et al., 2016; Xiao, Bu, & Shi, 2021).

In addition to the availability of shelter, the spatial distribution of freshwater turtles is closely related to the availability of food resources (Goodman & Stewart, 2000; Lindeman, 2003). Big‐headed turtles are omnivorous (Sung et al., 2016); thus, they require microhabitats that can provide suitable prey items. We found that big‐headed turtles prefer stream microhabitats with high species diversity. Their diet consisted of freshwater crabs (*Potamonautes hainanensis*), evidence of which was present in the feces of captured turtles, in addition to Venus fish (*Nicholsicypris normalis*), whitespotted clarias (*Clarias fuscus*), Vietnam catfish (*Silurus cochinchinensis*), and other fish that were found in the turtle cages. Food abundance may also influence the presence or absence of the big‐headed turtle. However, we did not quantify the number of individuals per potential prey species. Further studies are needed to examine the impact of food abundance on big‐headed turtle microhabitat selection to improve our understanding of this relationship. Moreover, the cages only contained animals as food items, and the plants in their diet require further research.

In this study, we found that the big‐headed turtle appears at 900–999 m elevations in the Diaoluo Mountain region, consistent with the survey results of Gong et al. (2006) conducted on Hainan Island. In the Yingge Ridge area of Hainan Tropical Rainforest National Park, however, we recently observed two big‐headed turtles at an elevation range of 283–423 m (Xiao, unpublished data). Some individuals were reported from areas with elevations ranging from 500 to 900 m, including areas in Guangdong Province (Wang, 2010). These records suggest that the minimum elevation selected by turtles in this area may be lower than that observed in the current study. It is worth noting that we made a more concentrated effort to identify big‐headed turtles within the elevation range of 900–999 m (61 cages), whereas there were too few cages in lower (<900 m; eight cages) and higher altitudes (≥1000 m; 12 cages), limiting the accuracy of the results. Therefore, future investigation should expand the scope to include the relationship between altitude and distribution of the big‐headed turtle in Hainan Tropical Rainforest National Park.

In summary, we found that big‐headed turtles on Diaoluo Mountain prefer stream microhabitats with rocky substrates, several caves, and high food diversity. These findings provide evidence to support the protection of rainforest habitat integrity and streams, thereby protecting the microhabitats and food sources important for big‐headed turtle survival and reproduction. In particular, our results can be used as a reference for the conservation of this species in Jianfeng Ridge, Yingge Ridge, and other areas in Hainan Tropical Rainforest National Park.

## AUTHOR CONTRIBUTIONS

**Fanrong Xiao:** Conceptualization (lead); data curation (lead); formal analysis (lead); funding acquisition (lead); methodology (lead); project administration (lead); resources (lead); software (lead); supervision (lead); validation (lead); writing – original draft (lead); writing – review and editing (lead). **Rongping Bu:** Data curation (equal); formal analysis (equal); writing – original draft (equal). **Zihao Ye:** Data curation (equal). **Jichao Wang:** Project administration (equal); writing – review and editing (equal). **Hai‐Tao Shi:** Conceptualization (equal); writing – review and editing (equal).

## CONFLICT OF INTEREST STATEMENT

The authors declare no conflicts of interest.

## Data Availability

The ecological factor data of the big‐headed turtle are available at: Dryad https://doi.org/10.5061/dryad.95x69p8px.
