# Correction to “Microhabitat characteristics of the critically endangered big‐headed turtle *Platysternon megacephalum*”

**DOI:** 10.1002/ece3.10773

**Published:** 2023-12-19

Xiao, F., Bu, R., Ye, Z., Wang, J., & Shi, H.‐T. (2023). Microhabitat characteristics of the critically endangered big‐headed turtle *Platysternon megacephalum*. *Ecology and Evolution*, 13, e10633.

In line 4 of the “Abstract” section, the text “across Southeast Asia.” was incorrect. This should have read: “across Asia.”

We apologize for this error.

